# Eosinophilic Esophagitis and Gastroesophageal Reflux Disease: An Overlapping of Clinical, Endoscopic and Manometric Features

**DOI:** 10.7759/cureus.15774

**Published:** 2021-06-20

**Authors:** Dimitrios Karapiperis, Carina Malmstrom, Spyridon Vrakas, Jonatan Gil, Simone Ignatova, Sara Elmahdy, Thomas Franzen

**Affiliations:** 1 Department of Gastroenterology, Vrinnevi General Hospital of Norrkoping, Norrkoping, SWE; 2 Department of Gastroenterology, Tzaneio General Hospital, Piraeus, GRC; 3 Department of Pathology, Linkoping University Hospital, Linkoping, SWE

**Keywords:** eosinophilic esophagitis, gastroesophageal reflux disease, high-resolution manometry, esophageal motility, steroids

## Abstract

The cause of eosinophilic esophagitis (EoE) is not well understood. Most patients with EoE have allergic disorders. Here, we describe a patient with gastroesophageal reflux and EoE with dysphagia, substernal discomfort and retrosternal pain. Based on symptomatology consistent with gastroesophageal reflux disease (GERD), treatment started with proton pump inhibitors (PPIs) but no effect was observed. Next, the patient underwent esophagogastroduodenoscopy and multiple biopsies were acquired from the lower and upper esophagus. Cortisone treatment was given and high-resolution manometry was performed before and after treatment. The results suggested that esophageal motility improved after cortisone therapy together with improvements in the clinical and histological pictures.

## Introduction

Eosinophilic esophagitis (EoE) is characterized by isolated eosinophilic infiltration in the esophageal mucosa. The pathogenesis of EoE remains unknown. EoE most commonly occurs in children and adolescents, with underlying allergic disorders, such as food allergy, atopic dermatitis, asthma, or allergic rhinitis [1]. Typically, symptoms improve with corticosteroid treatment. Food impaction and intermittent dysphagia are the most common symptoms in adult patients with EoE. Typical endoscopic findings include linear furrows, mucosal rings and white papules [2].

High-resolution manometry is becoming widely accepted in clinical practice for evaluating and categorizing esophageal motility disorders. The most frequent high-resolution manometry findings in EoE are early pan-esophageal pressurizations and weak peristalsis. Esophageal motility studies have also shown that patients with EoE had reduced distensibility and hypotonicity in the lower esophageal sphincter. These symptoms are also common in other esophageal motility disorders, such as achalasia and nutcracker esophagus [3].

Histopathologically, an EoE diagnosis is primarily based on eosinophil infiltration in the mucosa. However, other features might promote esophageal dysmotility. For example, increased fibroblast contractions have been observed in co-cultures of eosinophils and fibroblasts [4] and axonal necrosis has been described in EoE [5].

## Case presentation

A 25-year-old man visited our esophageal clinic with dysphagia, substernal discomfort and retrosternal pain, which had lasted for the past six months. He had allergic asthma and a history of animal, grass, and pollen allergies. Due to gastroesophageal reflux disease (GERD) symptomatology, the patient had been taking proton pump inhibitors (PPIs, 40 mg x 2) for the last three months but experienced no effect. 

**Figure 1:**
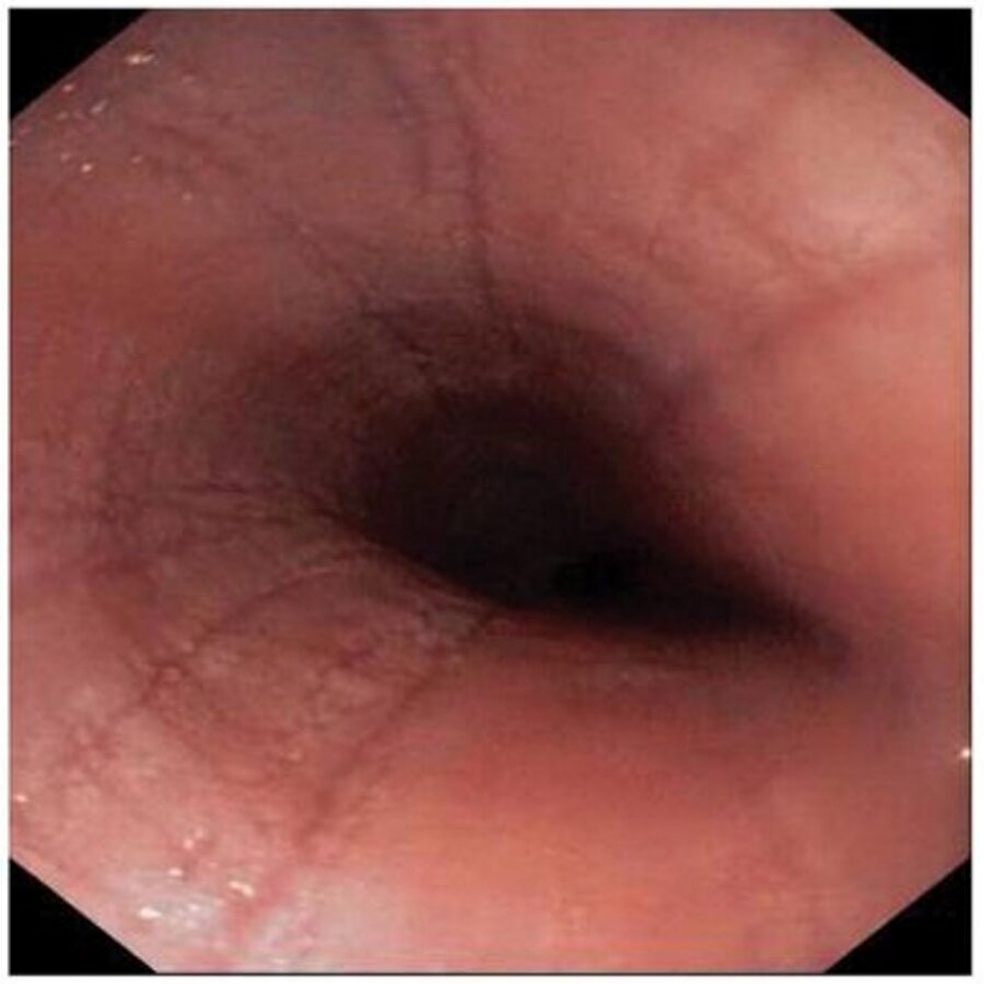
First endoscopy of the esophagus. Note the edema, linear furrows, and multiple mucosal nodularities.

An esophagogastroduodenoscopy showed linear furrows, edema of the mucosa and multiple nodularities in the upper and lower regions of the esophagus with grade C reflux esophagitis according to the Los Angeles classification system (Figure 1). Due to a suspicion of EoE, we acquired multiple biopsies from the lower and upper esophagus.

**Figure 2:**
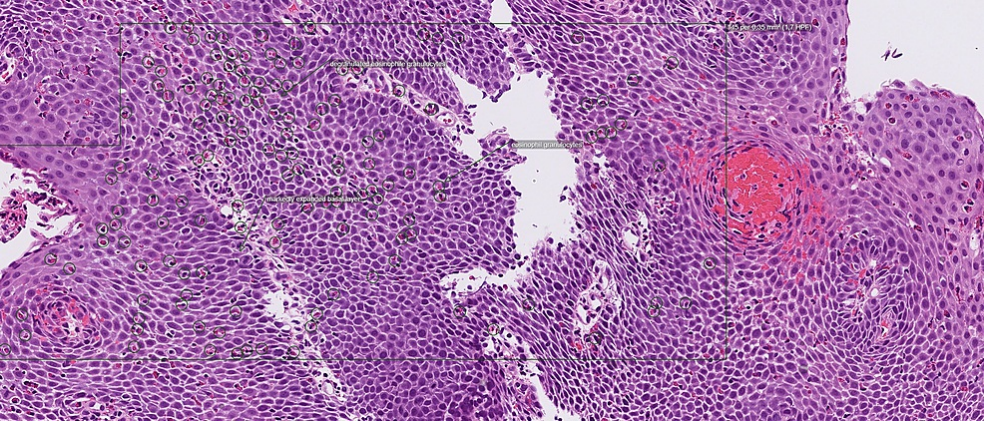
Histological analysis of a biopsy from the first esophageal endoscopy. Note the heavy eosinophilic infiltration in the esophageal mucosa.

Multiple biopsies were also taken from the stomach, bulb, and duodenum. The histopathological results from the duodenum and stomach were normal. However heavy eosinophilic infiltration was observed in the mucosa from esophagus with more than 145 eosinophils per high-power field (Figure 2). 

**Figure 3:**
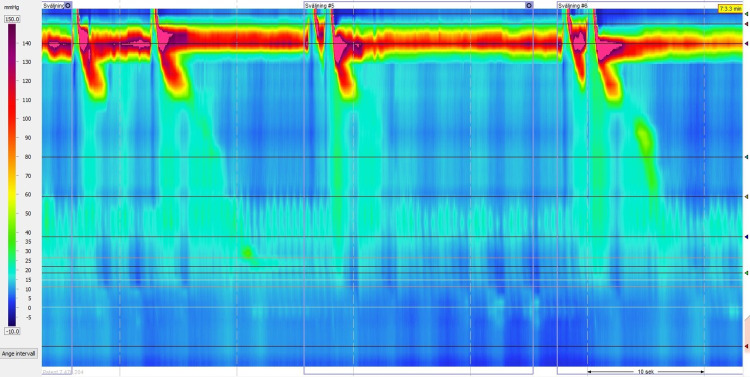
First high-resolution manometry plot shows intraluminal pressure of the esophagus. Note the esophageal motor skills are poor, weak, with a low distal contractile integral (DCI).

High-resolution manometry revealed a 5 cm gastrointestinal hernia with relatively low sphincter pressure and normal relaxation. The resting pressure (RP) was 7.2 mmHg (reference range: 13-43 mmHg) and the integrated relaxation pressure (IRP) was 3.5 mmHg (reference range: < 15 mmHg). At the upper esophageal sphincter, we observed normal sphincter pressure and a normal relaxation time. The esophageal motor skills were poor with a large number of failed swallows (70%). The remaining successful swallows (30%) were weak with a distal contractile integral (DCI) of 135.2 mmHg (reference range: 450-8000 mmHg) (Figure 3). 

Based on the endoscopic, histological and clinical findings, the patient was diagnosed with EoE. Due to the lack of response to PPI treatment and the patients’ allergies after the endoscopic examination, we started treatment with Budesonide tablets (2mg per day) and PPIs (40 mg x 2). 

**Figure 4:**
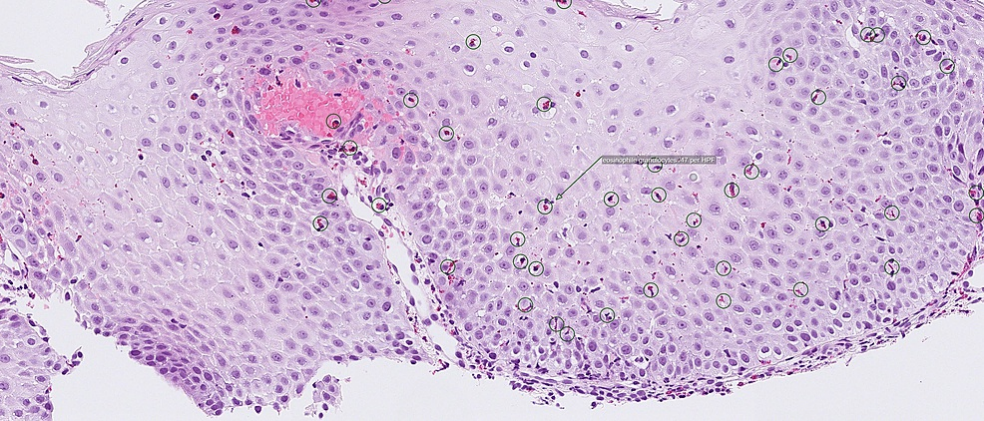
Histological analysis of a biopsy from the second esophageal endoscopy. Note the reduction in the eosinophil counts.

The patient’s symptoms improved gradually after starting treatment with steroids. Eight weeks after commencing treatment, a follow-up endoscopy revealed improvements in the edema, linear furrows, and mucosal irregularities but grade B esophagitis persisted. A mucosal biopsy from the second endoscopy showed a reduction in the eosinophil counts with 45 eosinophils per high-power field (Figure 4).

**Figure 5:**
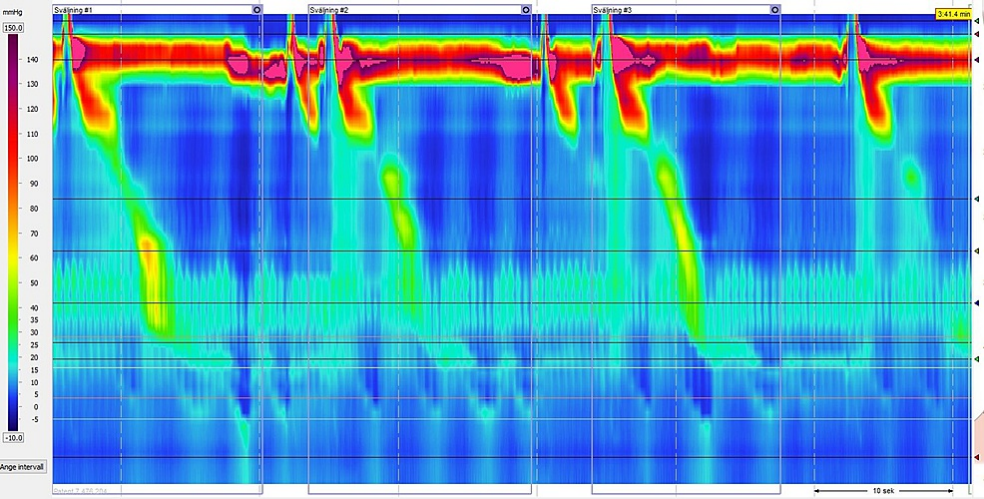
Second high-resolution manometry. Note the motor skills in the esophagus had changed. The peristalsis to the lower esophagus had not returned to normal but had substantially improved.

At the eight-week follow-up, high-resolution manometry showed the large hiatus 5-cm hernia. The esophageal sphincter was hypotonic but it showed good relaxation. The RP was 10.1 mmHg and the IRP was 4.2 mmHg. However, the motor skills in the esophagus had changed. Although the esophageal peristalsis had not returned to normal, it had substantially improved, compared to the first manometry readings. The DCI was 275.9 mmHg (Figure 5).

## Discussion

We described a patient that visited our clinic with dysphagia, substernal discomfort and pain. The patient had been treated for GERD but experienced no improvement in dysphagia. Therefore we performed endoscopy and found grade C esophagitis according to the LA classification system. The endoscopy showed lesions compatible with EoE. Histopathological analyses of multiple biopsies from the esophagus confirmed the coexistence of EoE and GERD. 

For the interaction between EoE and GERD, three scenarios have been proposed. Studies have suggested that GERD might be a risk factor for the development of EoE. Indeed, some studies have described abnormal acid reflux in patients with EoE. Remedios et al performed 24-hour esophageal PH monitoring in 24 patients with EoE and found that 10 (42%) had abnormal acid reflux [6]. In another study, Peterson et al performed 48-hour esophageal PH monitoring in 25 patients with EoE and found that 14 (56%) had abnormal acid reflux [7]. According to Spechler, GERD increases the permeability of the esophageal mucosa. Consequently, food antigens do not pass into the stomach; instead, they penetrate the mucosa of the esophagus and incite an allergic response, which contributes to the development of the syndrome [8]. TRPV1 (transient receptor potential cation channel) is activated by acid reflux. This activation has been linked to the release of inflammatory mediators and cytokines, including MIP-1a, eotaxin-1, eotaxin-2, eotaxin-3, IL-8, lyso-PAF AT, MCP-1, substance P, and calcitonin gene-related peptide (CGRP). Moreover, the activation of proteinase-activated receptor 2 (PARS2) in esophageal epithelial cells causes the production of IL-8. This immune response to gastric reflux material may contribute to the development of EoE [9]. Conversely, EoE might be a risk factor for the development of GERD. There are eosinophil products (IL-13, IL-6, vasoactive intestinal peptide and platelet-activating factor), that can relax the muscle of the lower esophageal sphincter that prevents reflux, and can alter esophageal motility to delay the clearance of refluxed acid [10]. The third scenario is that EoE and GERD coexist and there is no relationship. As mentioned, studies showed abnormal acid reflux during PH-monitoring in patients with EoE [6,7]. These data support an important interaction and not only the coexistence between EoE and GERD.

Dysphagia is a common symptom of EoE. Dysphagia is typically caused by esophageal stricture or a narrowing of the esophagus. However, some patients without strictures experience dysphagia. Thus, many authors have proposed that motility disorders might cause this symptom. Indeed, studies about motility disorders in patients with EoE describe a range of symptoms including absent peristalsis, achalasia, diffuse esophageal spasm, jackhammer esophagus, pan-esophageal pressurization and ineffective peristalsis [11]. In the present study, high-resolution manometry showed abnormal motility during swallowing. Our patient presented with a large proportion of failed swallows (70%) and the successful swallows (30%) were weak according to high-resolution manometry. 

There is evidence that eosinophils can release myoactive and neuroactive products in the esophageal mucosa which causes motility abnormalities. In patients with EoE eosinophils produce cytokines and growth factors that can affect esophageal smooth muscle contractility [12]. Eosinophils have been shown to secrete products that cause esophageal muscle contraction (thromboxane B2, prostaglandin F2, leukotriene D4) [13,14] and products that cause esophageal muscle relaxation (IL-13, IL-6, vasoactive intestinal peptide and platelet-activating factor)) [10-15]. In addition, mast cells might also have a significant role in the development of esophageal motility disorders in EoE patients. High numbers of mast cells have been observed in the esophageal mucosa with elevated cytokine expression [16]. Mast cells secrete proinflammatory agents such as TNF-a, TNF-b and tryptase which contribute to the formation of fibrosis. These cytokines also play an important role in collagen IV production [17,18].

Our patient did not respond to PPI therapy. An esophagogastroduodenoscopy showed linear furrows, edema of the mucosa and multiple nodularities in the upper and lower regions of the esophagus, compatible with eosinophilic esophagitis. We acquired multiple biopsies from the lower and upper esophagus and the coexistence of EoE and GERD was confirmed. Consequently, he was given a combination therapy of PPI (40 mg x 2) and topical cortisone with the aim of treating both GERD and EoE. Importantly this treatment improved esophageal motility and improvements were noted in the clinical and histological pictures. With high-resolution manometry, we observed that the esophageal peristalsis had not returned to normal but had substantially improved compared to the first manometry readings. Furthermore the patient reported symptom relief after the steroid treatment. Similarly, Savarino et al. described a patient with achalasia and EoE whose dysphagia disappeared with systemic prednisolone therapy. In addition that patient showed marked improvement in esophageal motility [19]. Lucendo et al. described nine patients with EoE and abnormal esophageal peristalsis. Those authors repeated manometry in seven patients after successful steroid treatment. Impressively all seven patients showed improvements in esophageal peristalsis after treatment [20]. Thus treating EoE appeared to improve the abnormalities detected with manometry.

## Conclusions

In conclusion, we described a patient with coexisting EoE and GERD and simultaneous esophageal motor disorders. These diseases may be more complex than originally thought and the interaction between them may depend more on individual patient characteristics. We found that EoE and motility disorder improved with the administration of topical corticosteroids. However, the patient did not show a complete response to the treatment; therefore, topical therapy is currently ongoing, and an elimination diet is planned. If the endoscopy after elimination diet shows histopathologically no eosinophilic infiltration but GERD, then an operation of the hiatus hernia has to be recommended. Future studies are needed to determine the interrelationship between eosinophilic infiltration, abnormal motility testing and dysphagia in patients with EoE and GERD.
